# Management of epistaxis in patients with ventricular assist device: a retrospective review

**DOI:** 10.1186/s40463-018-0295-6

**Published:** 2018-08-02

**Authors:** Clifford Scott Brown, Ralph Abi-Hachem, David Woojin Jang

**Affiliations:** Division of Head and Neck Surgery & Communication Sciences, Department of Surgery, Duke University Medical Center, DUMC 3805, Durham, NC 27710 USA

**Keywords:** Epistaxis, von Willebrand syndrome, Ventricular assist device, Anticoagulation

## Abstract

**Background:**

Patients with a ventricular assist device (VAD) are at risk for epistaxis due to the need for anticoagulation. Additionally, these patients develop acquired von Willebrand syndrome (AvWS) due to these devices. Management is complicated by the risk of thrombosis if anticoagulation is reversed. This study sought to characterize the clinical features and management of epistaxis in this high-risk population.

**Methods:**

Retrospective review of adults with VAD and epistaxis necessitating inpatient consultation with the otolaryngology service were included.

**Results:**

49 patients met inclusion criteria**.** All patients had a presumed diagnosis of AvWS. An elevated INR (> 2.0) was present in 18 patients (36.7%). Anticoagulation was held in 14 (28.6%) patients, though active correction was not necessary. Multiple encounters were required in 16 (32.7%) patients. Spontaneous epistaxis was associated with multiple encounters (*p* = 0.02). The use of hemostatic material was associated with a lower likelihood of bleeding recurrence (*p* = 0.05), whereas cauterization with silver nitrate alone was associated with a higher likelihood of re-intervention (p = 0.05). Surgery or embolization was not required urgently for any patient. Endoscopy under general anesthesia was performed for one patient electively. Mean follow up time was 16.6 months (σ = 6.3). At six months, 18 (36.7%) patients were deceased.

**Conclusion:**

While these patients are at risk for recurrent spontaneous epistaxis, nonsurgical treatment without active correction of INR or AvWS was largely successful. Placement of hemostatic material, as opposed to cautery with silver nitrate, should be considered as a first-line treatment in this group. Multidisciplinary collaboration is critical for successful management.

## Background

Epistaxis remains the most frequent otolaryngologic emergency and the second most common reason for referral to an otolaryngologist [1]. Over the years, many studies have sought to develop algorithms for treatment and prevention. Despite this, management of epistaxis remains a controversial and evolving topic. For example, there are no standard guidelines on duration of nasal packing and whether prophylactic antibiotics are necessary [2]. More recently, the advent of endoscopic sphenopalatine artery ligation (ESPAL) and vascular interventional procedures raises the question of when such interventions should be pursued [3].

Epistaxis in the setting of coagulopathy is difficult to manage, as patients often require management of systemic coagulopathy in addition to intranasal interventions. Regardless of whether patients are medically anticoagulated or suffer from an acquired or hereditary coagulopathy, they have longer average inpatient hospital stays and require more invasive measures of local hemorrhage control when they develop epistaxis [4]. The incidence is high, with 10–17% of patients developing epistaxis during long-term vitamin K antagonist therapy [5]. Given that there are 2.83 million quarterly visits with anticoagulation use in the United States, the at-risk population is significant [5–7].

One patient population that is especially at risk for epistaxis are those with ventricular assist devices (VAD). Patients with heart failure may receive a VAD as either a bridge to transplantation or as destination therapy. They often require multiple anti-coagulant and anti-platelet medications, with a goal international normalized ratio (INR) of 2.5 [8]. While bleeding is a major risk in these patients, sub-therapeutic levels can lead to devastating thromboembolic consequences, with a reported incidence of 2–47% [9]. Therefore, a delicate balance must be maintained. To make matters more complex, virtually all of these patients develop acquired von Willebrand syndrome (AvWS) as a result of the device itself. Patients with VAD are also poor surgical candidates due to their cardiac co-morbidities. All of the above factors create a complex situation when such a patient develops epistaxis.

Patients may develop von Willebrand disease (VWD) from a variety of etiologies, including hereditary and acquired origins. Given that VWD the most common hereditary blood-clotting disorder, its incidence is only increased by the multiple medical conditions that contribute to its development. On the most basic level, a deficiency in either the quantity or quality of von Willebrand factor (vWF) results in impaired platelet adhesion, and even in the mildest subtypes, epistaxis is a frequent symptom.

To our knowledge, this study would be the first to describe patient characteristics and management of epistaxis in this unique and complex patient population. We aimed to determine the efficacy of current treatment modalities as well as to identify risk factors for recurrence of epistaxis. Based on the findings of Smith et. Al [4], we hypothesized that patients with VAD would be more likely to require aggressive measures such as operative intervention.

## Methods

This is a retrospective review of adult patients (age greater than 18 years old) with an LVAD who required inpatient consultation with the otolaryngology service at Duke University Medical Center for epistaxis between July 1, 2006 and July 1, 2016. Institutional review board approval was obtained through Duke University. Patients were identified through the Duke Enterprise Data Unified Content Explorer (DEDUCE). All patients with LVAD were initially selected based on CPT code 0048 T or ICD codes V43.21 and Z95.811. From this group, those with a documented diagnosis of epistaxis (ICD codes 784.7 or R04.0) and associated otolaryngology inpatient consultation were selected.

Electronic medical records for these patients were then reviewed. Demographic information was recorded as well as information related to the LVAD (LVAD type, date of LVAD surgery). Furthermore, anticoagulant and antiplatelet medications, as well as vital signs and lab values at the time of consultation were extracted. Epistaxis was categorized as spontaneous versus traumatic. Additionally, the bleeding location, interventions performed, outpatient follow-up after discharge, and the outcomes of each intervention were extracted.

### Statistical analysis

Data were grouped categorically and analyzed with Pearson’s chi-squared test. Tests of independence assessed for correlations between data points. Statistical analyses were performed with SPSS 23.0 software (SPSS Inc., Chicago, IL), with *P* < 0.05 considered significant.

## Results

A total of 49 patients met the inclusion criteria, with 37 male and 12 female patients. Median age was 58 years (range: 18–85 years). The types of LVAD included 39 (79%) HeartMate II (HMII), seven HeartWare (14%), two Centimag (4%), and one VentrAssist (2%). The median time between LVAD placement and consultation for epistaxis was 46 days (range 2–2886). There were 15 (30%) patients who required consultation within 10 days of placement. Primary reasons for admission included cardiac symptoms (79.6%), epistaxis (12.2%), and other (8.2%). Forty-six patients were seen in an inpatient unit and three patients were seen in the emergency room.

**Fig. 1:**
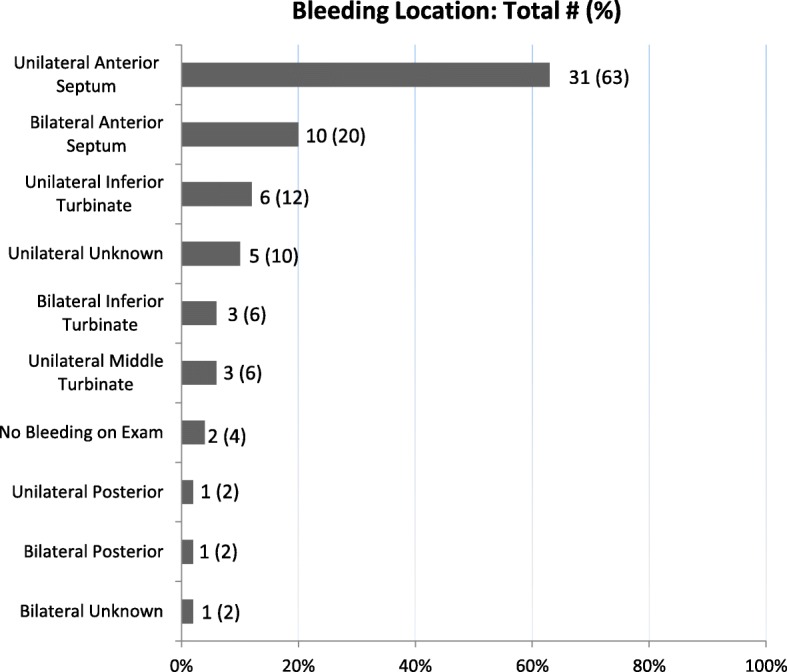
Bleeding Location: Distribution of bleeding locations by number and percentage. Note that sites are not mutually exclusive

Spontaneous non-traumatic bleeds occurred in 37 (75.5%) patients. The presence of spontaneous epistaxis was associated with multiple interventions (Chi-square = 5.345, *p*-value = 0.02). The most common site of bleeding was the anterior septum, with 31 (63.2%) patients bleeding from the unilateral septum, and 10 (20.4%) from the bilateral anterior septum (Fig. 1). Sixteen (32.7%) patients required multiple interventions from the otolaryngology service. Age, gender, and VAD type did not correlate significantly with spontaneous bleeding or the need for multiple interventions. Bleed location and the time between LVAD surgery and consultation did not correlate with spontaneous bleeding or need for multiple interventions. Mean follow-up time after initial consultation was 16.6 (σ = 6.3) months. 18 (36.7%) patients died prior to six month follow-up due to various causes, none of which were directly attributed to epistaxis. These findings are summarized in Table 1.

**Table 1:** Patient and Epistaxis Characteristics

Sex:	VAD Type:
Male: 37 (75%)	HMII: 39 (79%)
Female: 12 (25%)	HeartWare: 7 (14%)
Total: 49	CentriMag: 2 (4%)
	VentrAssist: 1 (2%)
Reason For Admission:	Cause:
Cardiac: 39 (79%)	Spontaneous: 37 (75%)
Epistaxis: 6 (12%)	Traumatic: 12 (25%)
Other: 4 (8%)	
Location:	Recurrence:
Anterior Septum: 41 (84%)	< 7 Days: 6 (12%)
Inferior Turbinate: 9 (18%)	7–30 Days: 6 (12%)
Other: 2 (4%)	1–6 Months: 4 (8%)
	> 6 Months: 28 (57%)
	Death < 6 Months: 18 (37%)

**Fig. 2:**
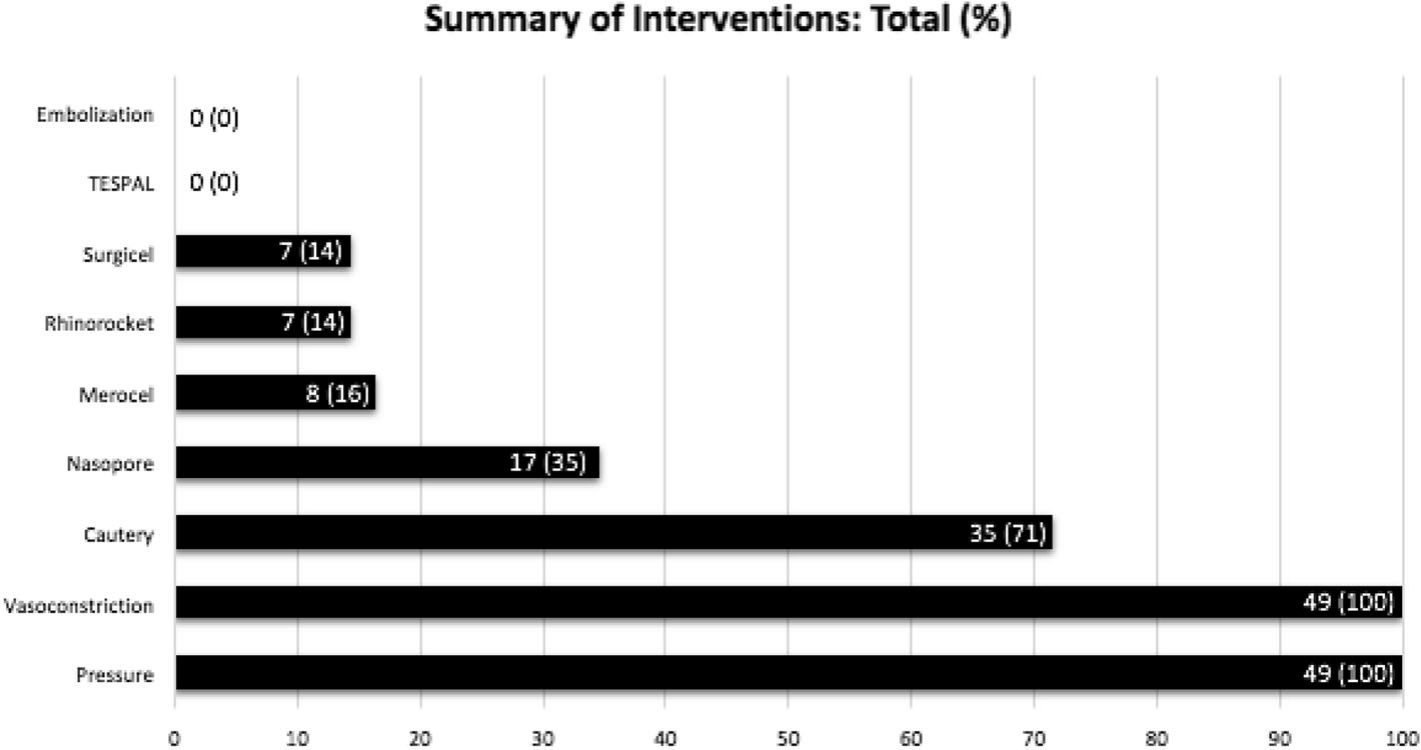
Summary of Interventions: Distribution of interventions performed by number and percentage. Note that interventions are not mutually exclusive

Each subject had an average of 1.59 (σ = 1.09) encounters. Interventions included use of oxymetazoline spray with application of direct pressure as an initial measure in all patients. Cauterization with silver nitrate was performed in 35 (71.4%) patients. The use of cautery alone was associated with a need for repeat interventions (Chi-square = 3.998, *p-*value 0.05). Dissolvable hemostatic material was used in 23 (46.9%) patients, with Surgicel (Ethicon, NJ, USA) used in 7 (14.3%) patients, and Nasopore (Stryker, MI, USA) used in 17 (34.7%) patients. Hemospore, which contains chitosan lactate, was not utilized. Non-dissolvable packing was used in 14 (28.6%) patients, with 8 (16.3%) patients receiving Merocel (Medtronic, MN, USA) and 7 (14.3%) receiving Rapid Rhino (ArthroCare, TX, USA). The use of dissolvable or non-dissolvable hemostatic material was associated with a lower likelihood of bleeding recurrence (Chi-square = 4.204, *p*-value = 0.04), however this effect was not significant when assessing either dissolvable and non-dissolvable packing alone. Conservative, or non-surgical, therapy was successful in all patients. There was a single patient who was taken to the operating room for an elective lysis of synechiae that had resulted from prior interventions, requiring general anesthesia secondary to the amount of scarring and prior failure of bedside attempts. Dissolvable packing was placed in the nose at the conclusion of the procedure. No patient required urgent operative intervention or angiography with embolization (Fig. 2).

All patients had an a priori diagnosis of AvWS as a result of the LVAD. Thirty-four patients (69.4%) were concurrently on warfarin, but only 18 (36.7%) of these had a therapeutic INR (between 2.0 and 3.0) at the time of epistaxis and only 4 (8.2%) had a supratherapeutic INR (greater than 3.0). Twenty-one (42.9%) patients were concurrently on heparin with 12 (24.5%) of these having prolonged partial thromboplastin time (greater than 40 s). Thirty-three (67.3%) patients were taking aspirin. Anticoagulant and antiplatelet medications were held in 14 (28.6%) patients because of epistaxis. Twenty-seven (55.1%) patients had normal platelet numbers (greater than 150,000) and 7 (14.3%) patients had platelet counts less than 100,000. No patient required active correction of anticoagulation with fresh frozen plasma, vitamin K, or protamine sulfate. There was no association between spontaneous bleeding or need for multiple interventions and the PTT, INR, or platelet count.

## Discussion

This study is the first to describe epistaxis management in patients with LVAD. Patients with LVAD represent a complex patient population due to the need for continuous anticoagulation and the development of AvWS. Moreover, these patients have end-stage heart failure and are poor candidates for general anesthesia.

In several ways, our results are consistent with the existing literature. The majority (75.5%) of our patients had spontaneous, non-traumatic epistaxis. This is consistent with the findings by Parajuli [10], who noted that 80–90% of general epistaxis patients had no identifiable cause. Also, 83.6% of episodes reported in our study occurred at the anterior nasal septum. This is consistent with the distribution seen in the general population, with literature reports of 80–90% of bleeds occurring at this location [1, 11]. Moreover, the presence of spontaneous epistaxis in our patients was associated with multiple interventions (*P* = 0.02), with 32.7% requiring multiple interventions. Similarly, Anghel et. Al [1] noted that patients with an idiopathic cause had the highest rate of recurrence over two years (26%). As for interventions, local cauterization with silver nitrate was performed in the majority of LVAD patients (71.4%). However, those undergoing cauterization alone without placement of hemostatic material were likely to require additional interventions at a later date. In their systematic review, Spielmann et al. noted that cautery was typically ineffective in patients receiving antiplatelet therapy [3].

In our study, the use of hemostatic material, both absorbable and non-absorbable, was associated with a lower likelihood of bleeding recurrence. However, there was no difference in efficacy amongst the type of material placed. This is consistent with existing literature demonstrating no significant difference in efficacy of various types of nasal packing [12, 13]. Moreover, there were no adverse events related to nasal packing in our study. Based on these findings, hemostatic material should be considered as initial management in patients with LVAD who present with epistaxis.

Recently, ESPAL has been shown to be associated with a reduction in cost and length of hospitalization in comparison to non-surgical treatment [14–17]. However, ESPAL should be considered carefully in patients with LVAD, given the high cardiac risks with general anesthesia and the bleeding risk from the surgery itself. For example, HeartMate II (HMII) devices have been associated with a significantly higher incidence of bleeding complications during surgical procedures [18]. In addition, the risk of postoperative infection is substantial in the LVAD population, as infection can seed the hardware [19]. Patients with LVAD who develop an infection have cumulative survival rates of 66.9% at 2 years compared to 81.3% for patients without an infection [20]. Although recent reviews suggest that patients with LVAD can safely undergo non-cardiac surgery [21], other studies showed that perioperative death occurred in a range of 6.4–16.7% [22]. Given these risks, surgery should be considered with caution in this patient population.

Because of the associated co-morbidities and need for anticoagulation with LVADs, a multi-disciplinary approach must be taken in management of epistaxis. In our study, holding anticoagulation was required in some patients, while it was continued in other instances. Interestingly, only 4 (8.2%) patients in this cohort had a supratherapeutic INR, and no association between spontaneous bleeding or multiple interventions with elevated INR was identified. This is consistent with the US-TRACE (STudy of Reduced Anti-Coagulation/Anti-platelEt therapy) study, which found that recurrent bleeding occurred in 52% of cases despite reduced antithrombotic therapy [23], suggesting alternative contributing factors to hemorrhagic complications.

Given the lack of association between INR and recurrent epistaxis, an important contributor may be AvWS, which results from the LVAD itself. LVADs produce continuous blood flow along an axial path using an internal rotor in the blood [24]. This results in the loss of large von Willebrand factor (vWF) multimers via a cleavage mechanism. In this situation, AvWS occurs immediately after implantation, and only resolves after device explantation [25, 26]. Serious bleeding, defined as episodes that result in death, reoperation, hospitalization, or transfusion, occurs in 19–40% of patients with the HMII, making it the most frequent complication [27], with the gastrointestinal tract and the nasal cavity being the most common sites [28]. The proteolytic mechanism that reduces VWF multimers also occurs in those with aortic valve stenosis, pancreatitis, liver cirrhosis, and leukemia [29]. According to the International Society on Thrombosis and Haemostasis, AvWS is most frequently associated with lymphoproliferative (48%), cardiovascular (21%), myeloproliferative (15%), other neoplastic (5%), and autoimmune disorders (2%) [30]. Importantly, AvWS must be distinguished from von Willebrand disease (VWD), an inherited disorder, due to different treatment approaches [31]. Diagnosis favors AvWS when patients have late onset of bleeding, typically after an uneventful surgery, along with an associated condition and a negative family history of bleeding. Desmopressin and VWF-containing concentrates are treatment options in VWD, but are ineffective in LVAD patients, with removal of the device being the only definitive treatment. Overall, AvWS is a condition that has implications beyond the LVAD population and should be recognized by otolaryngologists.

One limitation of this study is its retrospective nature. Another limitation is that we included only inpatient and emergency room consultations, which were mainly seen by residents of various training levels. Additionally, return visits to the emergency department that did not involve a consultation to otolaryngology were not included. However, we believe that our findings can be applied to care in the outpatient setting.

## Conclusion

Patients with LVAD present a unique challenge in the management of their epistaxis. Based on our findings, this difficulty stems from a variety of factors, including the need for anticoagulant or antiplatelet medication, development of irreversible AvWS, and high anesthetic risk. From the otolaryngologic perspective, use of hemostatic packing should be considered as a first-line intervention. Overall, a non-surgical multidisciplinary approach was successful in managing this complex patient population.

## Abbreviations

AvWS: Acquired von Willebrand syndrome
ESPAL: Endoscopic sphenopalatine artery ligation
HMII: HeartMate II
INR: International normalized ratio
PTT: Partial thromboplastin time
VAD: Ventricular assist device
VWD: Von Willebrand disease
VWF: Von Willebrand factor
